# Pulmonary Large-Cell Neuroendocrine Carcinoma: Molecular Heterogeneity, Current Treatment and the Emerging Precision-Oncology Paradigm

**DOI:** 10.3390/cancers18213585

**Published:** 2026-11-07

**Authors:** Tai-Huang Lee, Juei-Yang Ma, Huei-Yang Hung, Chia-Yu Kuo, Chin-Ling Chen, Pei-Chi Tsai, Cheng-Hao Chuang, Chih-Jen Yang

**Affiliations:** 1 Division of Pulmonary and Critical Care Medicine, Department of Internal Medicine, Kaohsiung Medical University Hospital, Kaohsiung Medical University, Kaohsiung 80708, Taiwan; 2 Division of Pulmonary and Critical Care Medicine, Department of Internal Medicine, Kaohsiung Gangshan Hospital, Kaohsiung Medical University, Kaohsiung 80708, Taiwan; 3 Department of Internal Medicine, Kaohsiung Municipal Siaogang Hospital, Kaohsiung 80708, Taiwan; 4 Cancer Center, Kaohsiung Medical University Hospital, Kaohsiung Medical University, Kaohsiung 80708, Taiwan; 5 School of Post-Baccalaureate Medicine, College of Medicine, Kaohsiung Medical University, Kaohsiung 80708, Taiwan

**Keywords:** LCNEC, neuroendocrine carcinoma, lung cancer, RB1, TP53, STK11, KEAP1, immunotherapy, DLL3, precision oncology

## Abstract

Pulmonary large-cell neuroendocrine carcinoma (LCNEC) is a rare, aggressive malignancy with substantial biological heterogeneity. Genomic studies identify SCLC-like and NSCLC-like spectra, but genomic categories overlap incompletely with transcriptional states. Morphology remains essential: neither neuroendocrine marker positivity nor RB1 status alone distinguishes LCNEC from small-cell lung cancer (SCLC). This narrative review integrates diagnostic limitations, stage-based management, and the clinical implications of profiling. Surgery with systematic nodal assessment is central for resectable disease; postoperative platinum chemotherapy has greater support than perioperative immunotherapy. In advanced disease, platinum-etoposide is a commonly used reference regimen. Retrospective associations involving platinum-gemcitabine or platinum-taxanes in RB1-wild-type tumors remain hypothesis-generating and should not be extended to pemetrexed. LANCE, a durvalumab phase II trial, and preliminary NEJ044 findings provide prospective chemo-immunotherapy observations, although randomized LCNEC-specific evidence remains absent. No standard second-line regimen is established. Rare actionable drivers may support matched treatment, whereas DLL3-directed approaches and FGL1/SPINK1-associated strategies require clinical validation. We propose an evidence-qualified framework separating stage-based care, individualized molecular considerations, and clinical-trial strategies.

## Simple Summary

Large-cell neuroendocrine carcinoma of the lung is a rare, aggressive cancer whose diagnosis and treatment are complicated by biological heterogeneity. This review links pathological assessment and molecular testing to specific clinical decisions while distinguishing routine stage-based care from investigational strategies. Surgery and platinum-based chemotherapy remain central. Small prospective studies support further evaluation of chemo-immunotherapy, but they do not establish randomized evidence of benefit. Molecular profiling can identify uncommon actionable alterations and clinical-trial opportunities; broad SCLC-like or NSCLC-like labels are not validated treatment-selection tests. We also address limited biopsy specimens, combined tumors, management after relapse, and priorities for prospective research.

## 1. Introduction

Pulmonary LCNEC was formally proposed as a distinct high-grade neuroendocrine carcinoma by Travis and colleagues in 1991, based on neuroendocrine architecture, large-cell cytology, high mitotic activity, necrosis, and neuroendocrine differentiation [1]. In the 2021 World Health Organization classification of thoracic tumors, LCNEC remains within the spectrum of pulmonary neuroendocrine neoplasms, and morphology remains the primary basis of diagnosis, supported by immunohistochemistry and, increasingly, molecular testing [2].

The central clinical problem is that LCNEC behaves aggressively but is not biologically uniform. Landmark sequencing studies demonstrated concurrent TP53/RB1 inactivation in some tumors and STK11, KEAP1, or KRAS alterations in others [3,4,5]. Biological diversity provides a rationale for testing different treatments; it does not itself prove that a molecularly selected regimen improves survival. This review complements broader disease overviews by organizing evidence around decisions: when to seek pathology review or additional tissue, what profiling can change now, and which treatment choices remain hypotheses. Its contribution is an evidence-qualified clinical synthesis, not a new validated classification or prediction model.

## 2. Methods and Literature Search Strategy

The original narrative review used a structured PubMed/MEDLINE search covering publications through August 2026. For this revision, targeted searches of PubMed-indexed records, publisher websites, ASCO abstracts, and trial registries were completed on 23 October 2026. The core concept was (“large cell neuroendocrine carcinoma” OR LCNEC) AND (lung OR pulmonary), combined with diagnosis, biopsy, RB1, molecular subtype, surgery, chemotherapy, immunotherapy, relapse, or radiotherapy. Named-study searches included LANCE, NEJ044, LOGIK2401/NECTAR, and JCOG1205/1206. Reference lists and DOI-linked bibliographic records were used for supplementary identification and verification.

Eligible material addressed pulmonary LCNEC diagnosis, biology, treatment, or outcomes. Mixed-histology studies were retained when their LCNEC relevance could be specified; extrapulmonary neuroendocrine studies were not treated as direct lung evidence. Prospective intervention studies were distinguished from prospective observational cohorts, retrospective comparisons, pooled observational analyses, and preclinical work. Conference abstracts were included for important emerging data but explicitly labeled preliminary. SCLC and NSCLC findings were used only with a stated extrapolation caveat.

Evidence was selected for clinical relevance, diagnostic ascertainment, study design, and applicability to the treatment setting. Where possible, original reports were favored over secondary summaries. This was not a systematic review: no preregistered protocol, duplicate-screening record, or formal risk-of-bias assessment is reported, and no quantitative pooling was performed. Consequently, completeness and freedom from selection bias cannot be assumed. The March 2026 systematic review of prospective interventional trials provides a complementary assessment with different eligibility criteria and an earlier evidence cutoff [6].

## 3. Epidemiology and Clinical Characteristics

LCNEC is uncommon. A Surveillance, Epidemiology, and End Results (SEER) analysis reported that LCNEC represented 0.58% of lung and bronchial carcinomas, while another SEER study estimated an age-adjusted incidence of approximately 0.3 per 100,000 persons [7,8,9,10]. Patients are typically older adults, frequently men, and most cohorts show a strong association with tobacco exposure. Upper-lobe and peripheral tumors are common, and advanced-stage disease is frequent at presentation [7,8,11].

Population-based survival remains poor. In the SEER analysis by Deng et al., 5-year overall survival (OS) was 16.7% and lung cancer-specific survival was 20.7% [8]. These estimates are strongly stage dependent and should not be applied directly to carefully selected resected cohorts. Table 1 summarizes the epidemiologic and clinical profile.

## 4. Pathology and Diagnosis

LCNEC is a morphologic diagnosis supported by neuroendocrine immunohistochemistry (IHC). Features include organoid nesting, trabeculae, palisading or rosettes; necrosis; more than 10 mitoses per 2 mm^2^; and non-small-cell cytology with appreciable cytoplasm and conspicuous nucleoli [1,2]. Synaptophysin, chromogranin A, CD56, and INSM1 support neuroendocrine differentiation. A single positive marker may support a morphologically convincing diagnosis, but isolated positivity, particularly nonspecific CD56 staining, is insufficient without compatible architecture and cytology. Ki-67 is useful in difficult small specimens but is not the principal WHO criterion distinguishing pulmonary LCNEC from SCLC [2].

**Figure 1:**
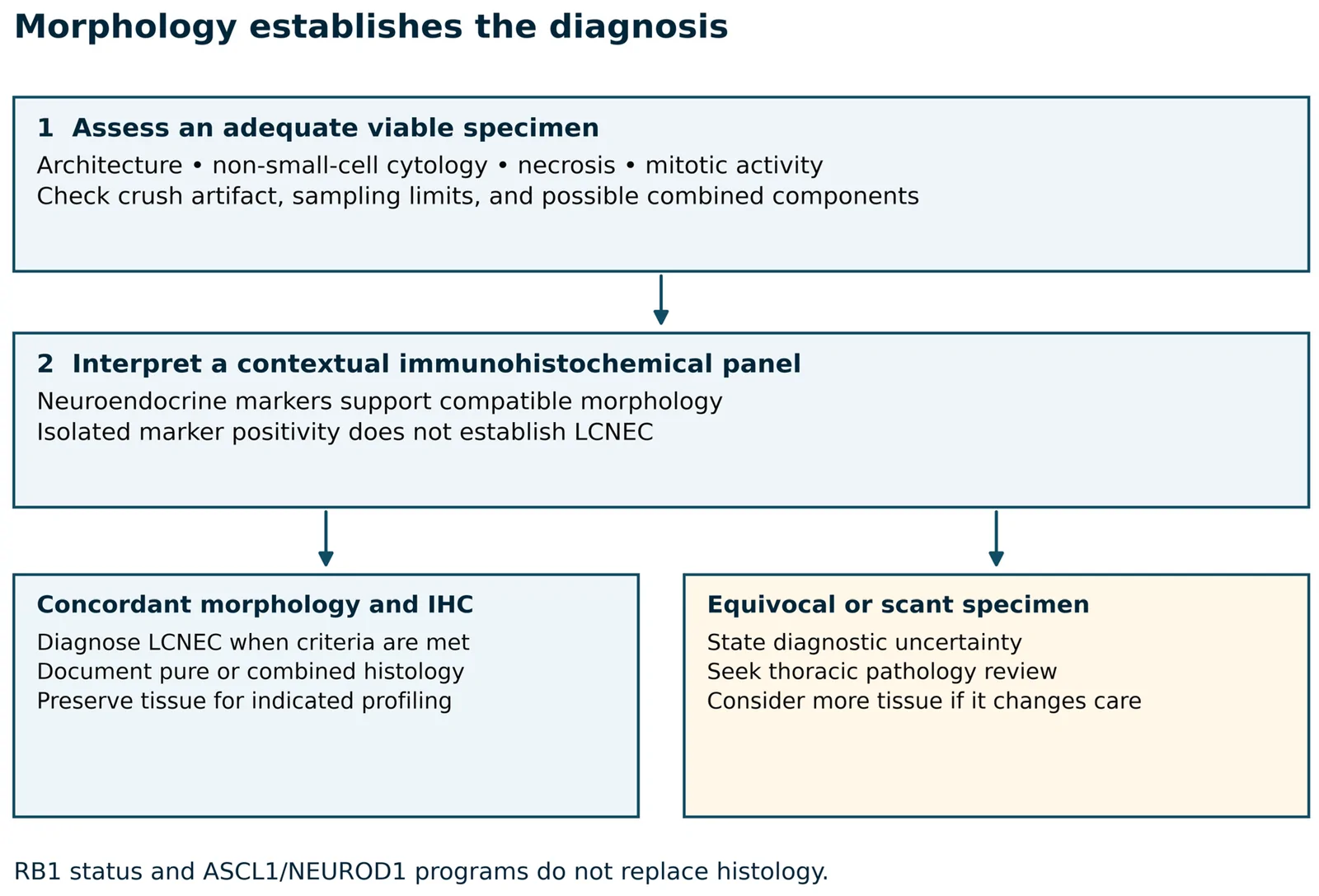
Diagnostic assessment of suspected pulmonary LCNEC. Morphology and a contextual immunohistochemical panel are interpreted together. An uncertain small specimen triggers review or additional sampling rather than an automatic LCNEC diagnosis. Combined components and sampling limitations are documented. Original schematic prepared for this revision; scientific basis: [1,2,15,16]. LCNEC, large-cell neuroendocrine carcinoma.

Small biopsies and cytological preparations may lack architecture and contain crush artifact, necrosis, or too few viable cells for reliable classification. A resection can reveal LCNEC or a combined component missed on the initial biopsy; conversely, central review may reclassify an apparent LCNEC as SCLC or another poorly differentiated carcinoma [15,16]. Diagnostic certainty should therefore be stated rather than inferred from an immunostain panel. In equivocal cases, expert thoracic pathology review, comparison with prior specimens, and additional sampling should be considered when feasible and likely to alter management. Figure 1 incorporates this diagnostic uncertainty.

Combined LCNEC contains a morphologically distinct non-neuroendocrine component, such as adenocarcinoma or squamous carcinoma. Tumors containing a small-cell component require assessment under the combined-SCLC criteria [2]. Limited sampling can conceal either component and distort apparent genotype–histology associations. Studies should therefore report specimen type, pure versus combined histology, diagnostic criteria, and central review. Cohorts based solely on registry coding are not diagnostically equivalent to centrally reviewed trials, and reclassification can change both the evaluable denominator and apparent treatment response [15,16].

ASCL1-high and NEUROD1-high states describe neuroendocrine lineage programs, not separate morphological diagnoses. Both LCNEC and SCLC can express these transcription factors and conventional neuroendocrine markers. SCLC more typically shows scant cytoplasm, nuclear molding, finely granular chromatin, and inconspicuous nucleoli; LCNEC has non-small-cell cytology, although overlap and artifact limit reproducibility [2,3,4]. Shared TP53/RB1 alterations or an SCLC-like expression profile cannot override convincing LCNEC morphology.

Tissue stewardship is therefore part of diagnosis: an appropriately focused immunohistochemical panel should answer the differential diagnosis while preserving material for clinically useful sequencing. Additional stains are not a substitute for adequate morphology. When tissue is exhausted, the report should distinguish a confirmed LCNEC diagnosis from a provisional high-grade neuroendocrine carcinoma designation; that uncertainty should remain visible in clinical datasets and treatment discussions [2,16].

## 5. Molecular Heterogeneity

Rekhtman et al. demonstrated two major genomic subsets. SCLC-like tumors showed TP53/RB1 co-alteration, whereas NSCLC-like tumors lacked the canonical TP53/RB1 combination and instead were enriched for STK11, KEAP1, and KRAS. In that cohort, TP53, RB1, STK11, KEAP1, and KRAS alterations occurred in 78%, 38%, 33%, 31%, and 22%, respectively [3].

George et al. expanded this concept using integrated genomic and transcriptomic profiling. Type I LCNEC was enriched for TP53 together with STK11/KEAP1 and showed an ASCL1-high/DLL3-high/NOTCH-low neuroendocrine phenotype, whereas Type II LCNEC was enriched for TP53/RB1 inactivation but had lower neuroendocrine-marker expression and greater immune-related transcription [4]. Thus, genomic resemblance to SCLC does not automatically imply an identical transcriptional state.

**Figure 2:**
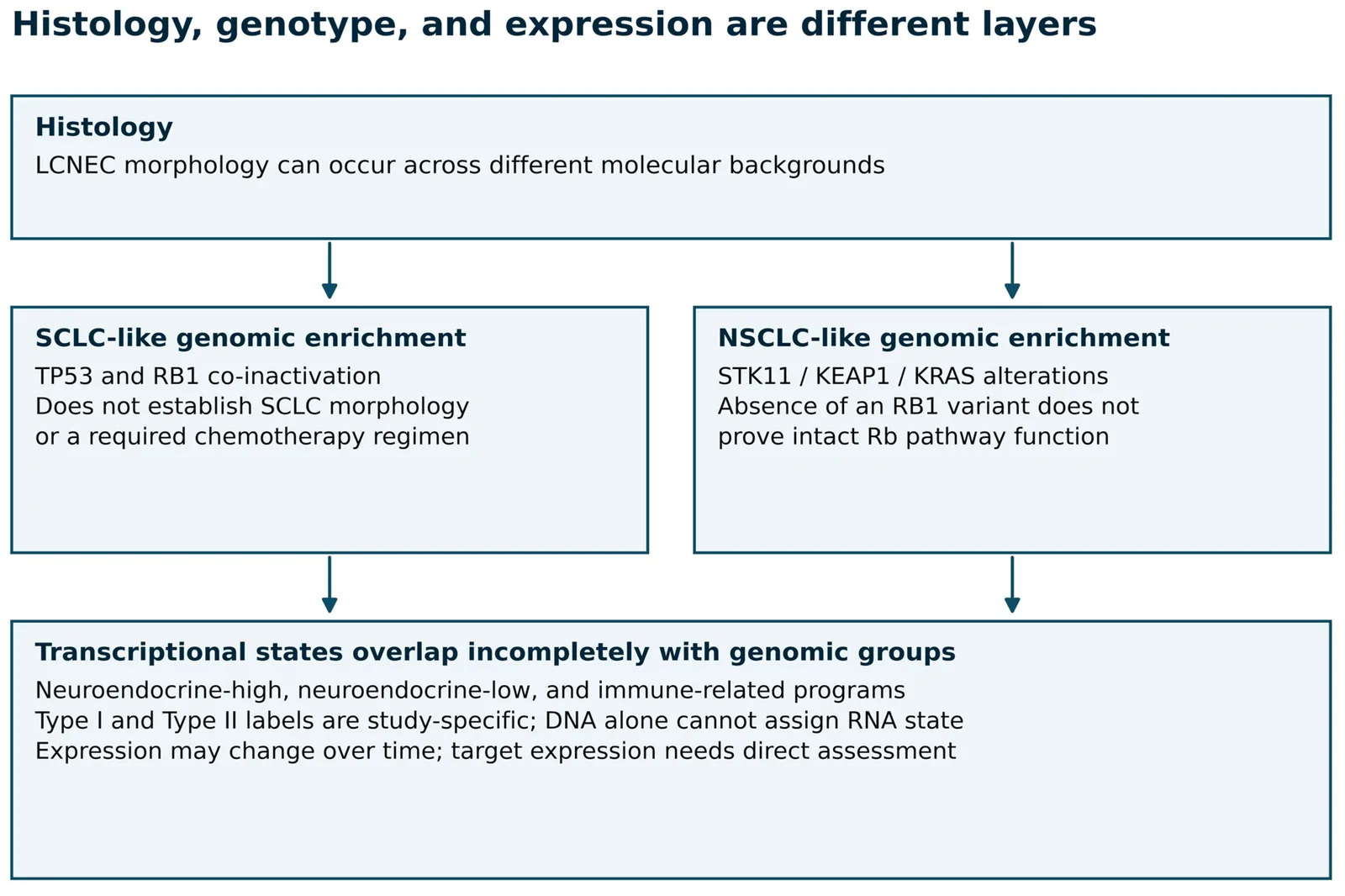
Distinct levels of LCNEC classification. Histology, genomic alterations, and transcriptional state provide related but non-equivalent information. The illustrated patterns are enrichments rather than mutually exclusive diagnostic rules or validated predictive biomarkers. Original schematic prepared for this revision; scientific basis: [3,4,5,14,17].

Miyoshi et al. confirmed recurrent TP53/RB1 alterations and less frequent potentially targetable pathway changes [5]. An integrated 2025 analysis combined clinical and molecular cohorts, identifying subtype-associated DLL3, FGL1, and SPINK1 expression and temporal changes in transcriptional states [14]. These observations motivate research rather than establish subtype-specific treatment efficacy. Figure 2 distinguishes the different classification levels.

RB1-wild-type SCLC is an important exception to binary classification. An integrated genomic/IHC study identified a small Rb-proficient subset with frequent combined histology and enrichment for neuroendocrine-low programs [17]. Absence of a detected RB1 mutation is not synonymous with intact Rb function: sequencing can miss structural or splice alterations, and expressed protein can be nonfunctional. Rb IHC, assay coverage, broader genomic findings, and morphology should be interpreted together. Neither RB1 wild-type nor STK11/KEAP1/KRAS alterations alone establish NSCLC-like LCNEC or exclude SCLC.

Similar LCNEC morphology despite different genomic profiles may reflect convergence on neuroendocrine differentiation and proliferative programs through distinct upstream alterations. ASCL1/NOTCH regulation, epigenetic state, cell of origin, and lineage plasticity are plausible contributors rather than a proven single mechanism [3,4]. Genomic categories describe alterations, whereas transcriptional categories describe the expressed cellular state. George et al. linked STK11/KEAP1-enriched Type I tumors to strong neuroendocrine expression and TP53/RB1-enriched Type II tumors to weaker neuroendocrine and greater immune-related expression [4]. These labels are therefore not interchangeable with a simple SCLC-like versus NSCLC-like treatment rule.

For limited specimens, low tumor fraction, assay coverage, and spatial heterogeneity can yield incomplete or apparently discordant classifications. DNA sequencing alone does not establish an RNA-defined state, and a biopsy may not represent all tumor components. Inadequate results should be reported as unclassified, not forced into a binary subgroup. Treatment should not be delayed solely to obtain an investigational subtype assignment.

## 6. Treatment Strategy

### 6.1. Resectable Disease

For medically fit patients with resectable LCNEC, surgery is the cornerstone of treatment. A National Cancer Database (NCDB) analysis of 6092 patients with stage I–IIIA disease associated surgery with favorable stage-specific outcomes and perioperative chemotherapy with better survival in pathological stage II–IIIA disease [18]. Lobectomy is generally favored when pulmonary reserve permits; retrospective comparisons do not establish oncologic equivalence of sublobar resection. Segmentectomy or wedge resection may be necessary for limited reserve, but margins and nodal evaluation remain important. Systematic hilar and mediastinal node assessment improves staging and informs postoperative treatment; clinical N0 status does not exclude occult nodal disease.

Evidence for postoperative chemotherapy is stronger beyond stage IA. A prospective cisplatin-etoposide study included only 15 patients, and a national analysis associated adjuvant chemotherapy with improved OS in stage IB but not stage IA disease [19,20]. JCOG1205/1206 randomized patients with completely resected high-grade pulmonary neuroendocrine carcinoma, including LCNEC and SCLC, to cisplatin-etoposide or cisplatin-irinotecan; irinotecan did not demonstrate superiority [21]. Updated survival results support that interpretation [22]. This mixed-histology randomized trial informs regimen choice but does not establish chemotherapy benefit over observation in LCNEC or justify describing all LCNEC treatment evidence as nonrandomized.

For selected potentially resectable N2 disease, invasive mediastinal staging and multidisciplinary review are essential. Surgery may be considered within a multimodality strategy when complete resection appears feasible, but LCNEC-specific evidence does not define the optimal selection criteria or sequence. Bulky or multistation N2 disease should not be assigned to surgery from histology alone. Neoadjuvant chemotherapy remains individualized, and no prospective LCNEC evidence establishes a preferred neoadjuvant regimen. Perioperative immunotherapy is less established than postoperative platinum chemotherapy and should not be assumed effective from NSCLC trials.

For medically inoperable, node-negative early-stage disease, stereotactic body radiotherapy (SBRT) offers a local-treatment option. LCNEC-specific comparisons are retrospective and confounded by operability and comorbidity [23]. SBRT does not provide pathological nodal staging, and whether additional systemic therapy improves outcomes is unresolved. These limitations should be discussed when balancing local control, systemic relapse risk, and treatment tolerance.

Postoperative decisions should incorporate pathological stage, resection completeness, nodal status, renal function, hearing, neuropathy, and recovery from surgery. The stage IA evidence does not prove that chemotherapy is ineffective in every individual; it indicates that routine benefit has not been established in that population. Conversely, an observational stage IB association is not equivalent to a randomized estimate of absolute benefit. Patients should be informed of the uncertainty when the anticipated toxicity is substantial. Nodal involvement increases concern for systemic relapse and strengthens the rationale for postoperative systemic treatment, while the role of postoperative radiotherapy requires a separate assessment of margins and locoregional risk [18,19,20].

### 6.2. Locally Advanced Disease

No randomized LCNEC-specific trial defines treatment for unresectable stage III disease. In an NCDB analysis of 5797 patients, chemoradiotherapy was associated with median OS of 16.1 months versus 11.9 months with chemotherapy alone [24]. Smaller retrospective series also report activity with platinum-etoposide and thoracic radiotherapy [25,26]. These associations are vulnerable to confounding by performance status, disease burden, and treatment selection; they do not establish the superiority of concurrent over sequential treatment. Definitive chemoradiotherapy may be considered for selected fit patients after multidisciplinary assessment, with concurrent treatment extrapolated from other lung cancers. The chemotherapy backbone, radiation dose/fractionation, and sequencing remain undefined in LCNEC. Consolidation durvalumab is likewise an extrapolated, individualized option rather than an established LCNEC standard.

### 6.3. Advanced and Metastatic Disease

Platinum-etoposide is a commonly used first-line reference regimen. GFPC 0302 prospectively evaluated cisplatin-etoposide, while a separate phase II study evaluated cisplatin-irinotecan [15,27]. Both support chemotherapy activity but lack randomized comparisons in advanced LCNEC. Central pathology review and differences between enrolled and confirmed LCNEC populations complicate cross-trial interpretation. Retrospective series add clinical experience but cannot reliably identify the optimal doublet [28,29].

Derks et al. associated RB1-wild-type disease with better outcomes on platinum-gemcitabine or platinum-taxanes than platinum-etoposide [30]. This finding concerns those specific regimens, not all NSCLC doublets. A separate pathology-reviewed cohort reported less favorable outcomes with platinum-pemetrexed [31]; pemetrexed should therefore not be substituted under a generic “NSCLC-type” label. The retrospective subtype association is hypothesis-generating, subject to selection bias and small subgroup sizes, and lacks prospective predictive validation. RB1 status alone should not mandate a backbone switch.

### 6.4. Immunotherapy

PD-L1 expression is heterogeneous; a surgical series found tumor-cell positivity in 22.3%, with different prognostic associations for tumor-cell and immune-cell staining [32]. This does not establish a predictive threshold for advanced LCNEC. Evidence for immune-checkpoint inhibitors (ICIs) should be separated by design rather than equating a large pooled population with randomized evidence.

Prospective evidence is available. LANCE enrolled 17 patients nonrandomly: 10 received atezolizumab plus carboplatin-etoposide and seven chemotherapy alone. Twelve-month OS was 57.1% versus 14.3%, but very small groups and nonrandom allocation preclude a definitive causal estimate [33]. Dudnik et al. treated 12 patients with durvalumab plus platinum-etoposide in a single-arm phase II trial that closed before its planned enrollment of 22. The objective response rate (ORR) was 50%, median progression-free survival (PFS) 4.4 months, and median OS 12.8 months; the 12-month PFS estimate was 25.0% (95% CI, 6.0–50.5). Early closure and the wide interval make these descriptive findings rather than confirmation of the prespecified hypothesis [34].

NEJ044, presented at ASCO 2026, prospectively observed atezolizumab-containing regimens across multiple institutions. The abstract reported 77 enrolled patients, 76 treated, and approximately 70% 1-year OS [35]. Chemotherapy partners varied, including etoposide and taxane-based combinations; the study cannot isolate the effect of one backbone or establish comparative efficacy. These conference findings remain preliminary pending full publication. The March 2026 systematic review identified four eligible prospective interventional studies under its criteria [6]; its conclusions should not be conflated with a later inventory that includes prospective observational studies or subsequent publications.

Tsukada et al. pooled 22 studies comprising 3537 patients. Chemo-immunotherapy had a pooled ORR of 49% and was associated with better PFS (hazard ratio [HR], 0.51) and first-line OS (HR, 0.72) than chemotherapy alone [36]. These pooled associations remain vulnerable to heterogeneous regimens, diagnostic criteria, treatment selection, and residual confounding. The absence of a statistically significant increase in severe adverse events does not prove equivalent safety.

In contrast, the integrated 2025 study of 590 patients did not demonstrate clear OS separation among systemic-treatment groups [14]. Differences in eligibility, treatment era, confounding adjustment, and pooling may contribute to the apparently discordant findings; neither analysis proves that ICIs benefit all LCNEC subgroups or that they lack benefit. The meta-analysis increases precision around observational associations without removing bias, whereas the molecular study mainly informs biological heterogeneity. Together with the small prospective studies, these data support individualized discussion and further trials rather than a definitive comparative standard. LOGIK2401/NECTAR is a single-arm phase II study of durvalumab plus carboplatin-etoposide with a planned sample size of 30. Its registry listed recruitment as ongoing when checked on 23 October 2026; 30 is a target, not confirmed accrual [37,38].

### 6.5. DLL3 and Emerging Targets

DLL3 expression was reported in 74% of stage IV LCNEC specimens in one series [39]. Expression provides a rationale for drug development, but does not establish a response threshold or clinical efficacy. The integrated molecular study linked DLL3 and FGL1/SPINK1 expression to different genomic spectra [14]. Such enrichment is cohort-dependent and should not replace direct target assessment for trial eligibility.

DLL3-directed T-cell engagers have a therapeutic rationale, but LCNEC-specific prospective validation is required before routine use can be inferred from SCLC. FGL1-associated immune suppression through the LAG-3 axis and SPINK1-associated biology are earlier-stage hypotheses. Biological expression, pharmacological targetability, and demonstrated patient benefit are separate evidentiary steps; neither FGL1 nor SPINK1 currently defines a validated LCNEC treatment-selection test.

### 6.6. Targeted Therapy and Genomic Profiling

Broad next-generation sequencing can identify uncommon actionable alterations, particularly in never-smokers, younger patients, or tumors with an adenocarcinoma component [3,5,14]. A detected alteration must be evaluated for pathogenicity and drug-specific actionability: an unspecified BRAF or ERBB2 alteration is not automatically equivalent to a validated target, and PI3K-pathway alterations do not establish an effective matched therapy in LCNEC. For a validated driver, molecular tumor board review and an appropriate basket trial are preferable; treatment extrapolated from NSCLC requires acknowledgment of sparse LCNEC-specific efficacy data. Table 2 and Table 3 summarize treatment evidence and biomarker limitations.

### 6.7. Relapsed or Refractory Disease

No established standard second-line regimen exists for pulmonary LCNEC. Choice depends on the prior regimen, response duration, performance status, organ function, residual toxicity, and patient priorities. Trial referral should be considered early. Rebiopsy may clarify combined histology, transformation, or a newly actionable finding when the result could change treatment; it is not mandatory for every progression.

A retrospective series included 39 patients with LCNEC or possible LCNEC receiving second-line chemotherapy, most commonly amrubicin. ORR was 7.7%, median PFS 3.3 months, and median OS 8.3 months [40]. These results illustrate limited activity and the diagnostic heterogeneity of salvage cohorts. Amrubicin, where available, irinotecan, or docetaxel may be considered using small-series evidence; no comparison establishes a preferred sequence. Platinum rechallenge after a durable prior response is an individualized extrapolation, without a validated LCNEC-specific treatment-free interval. Topotecan or lurbinectedin use derives mainly from SCLC experience and should not be presented as an LCNEC standard. Similarly, ICI rechallenge after progression on chemo-immunotherapy lacks established benefit. Later-line care should integrate symptom-directed radiotherapy and supportive care, with further systemic therapy proportionate to likely benefit and toxicity.

## 7. CNS Metastasis and Surveillance

LCNEC has a clinically important propensity for central nervous system (CNS) dissemination. Population-based data found brain metastases at presentation in approximately 19% of LCNEC, while a stage IV institutional series reported brain involvement in 47% during the disease course [10,12]. In a curatively treated cohort, the cumulative incidence of brain recurrence was 21.4% at 1 year and 41.3% at 2 years, with brain metastasis frequently occurring as a first relapse [13]. Similarly, an analysis of unresected stage III-IV high-grade neuroendocrine carcinomas reported substantial brain recurrence in LCNEC in the absence of prophylactic cranial irradiation (PCI) [25]. These observations support a low threshold for baseline brain MRI in advanced or locally advanced disease and consideration of MRI-based CNS surveillance during follow-up, although the optimal surveillance interval has not been prospectively defined. 

Routine PCI is not supported by randomized LCNEC-specific evidence. Retrospective data have suggested possible benefit, including a small series in which PCI was associated with numerically longer progression-free and overall survival, but these findings are highly susceptible to selection bias [26]. Therefore, PCI should not be automatically extrapolated from SCLC and should be individualized, preferably in a multidisciplinary setting or clinical trial. When brain metastases are detected, treatment should follow contemporary principles based on number, size, symptoms, resectability, extracranial disease control, and performance status. Retrospective LCNEC-specific datasets support the feasibility of stereotactic radiosurgery (SRS) for selected patients: an NCDB analysis associated upfront SRS with longer survival than whole-brain radiotherapy, and a Japanese multicenter series also demonstrated clinically meaningful outcomes after Gamma Knife radiosurgery [41,42]. A recent tertiary-center cohort further confirmed the poor prognosis after CNS involvement and the use of surgery, SRS, and whole-brain radiotherapy in real-world practice [43].

## 8. Practical Precision-Oncology Framework

A practical framework begins with diagnostic confidence, stage, resectability, functional status, and patient preferences. Its clinical value lies in identifying specific consequences of testing: expert review can correct an uncertain diagnosis; recognition of a combined component can prompt broader profiling; and a validated actionable alteration can support matched-treatment discussion or trial referral. When profiling reveals only TP53/RB1 or STK11/KEAP1/KRAS patterns, it usually supplies biological context rather than a mandatory treatment decision. Platinum-etoposide remains a reference option, with platinum-gemcitabine/taxanes considered individually in selected cases and chemo-immunotherapy discussed using its nonrandomized evidence base [30,33,34,35,36].

**Figure 3:**
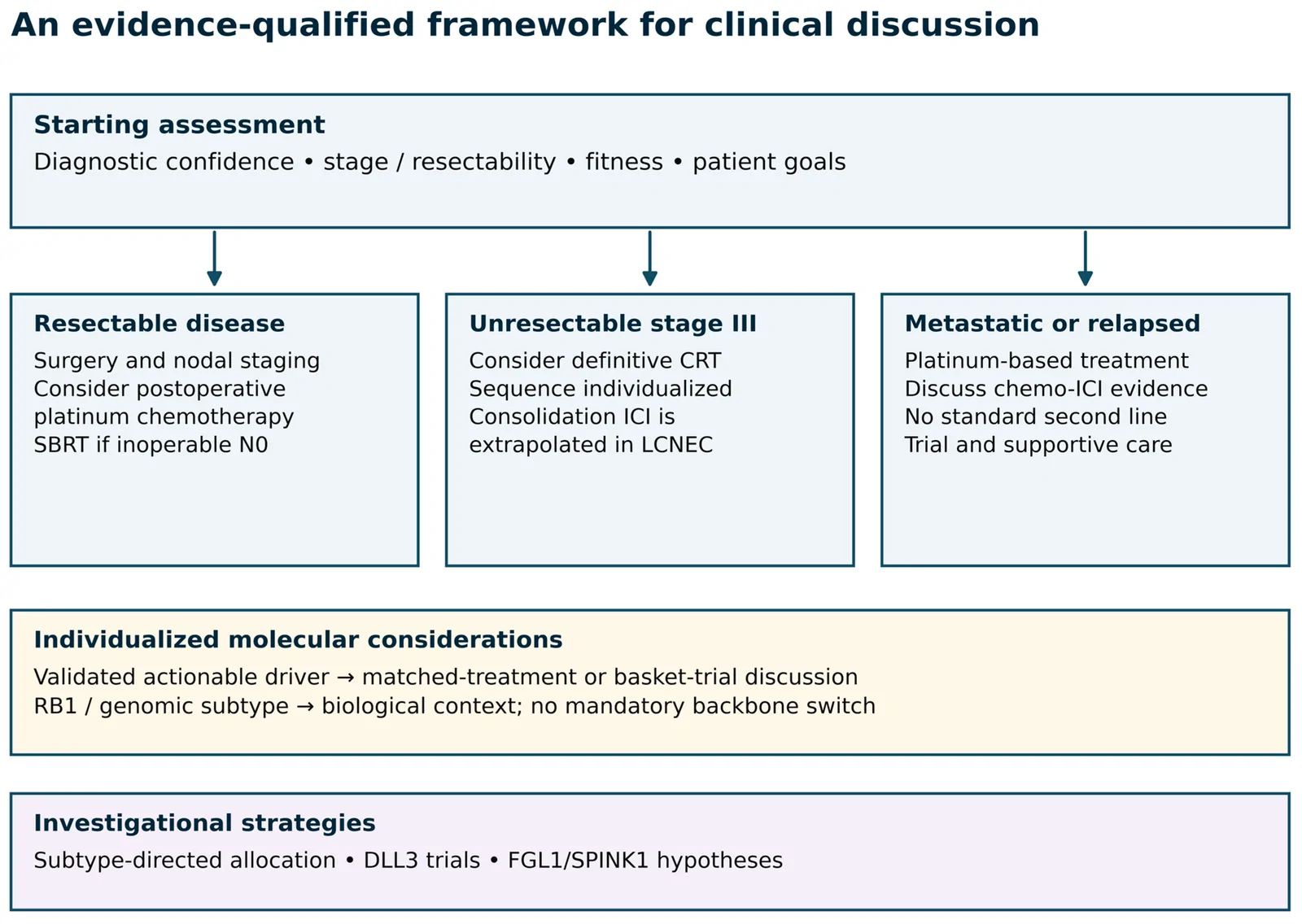
Evidence-qualified treatment framework. Stage-based management is separated from individualized molecular considerations and investigational approaches. No branch mandates chemotherapy selection from RB1 status or an SCLC-like/NSCLC-like label. The evidence remains limited even for commonly used clinical approaches. Original schematic prepared for this revision; scientific basis: [6,15,18,19,20,21,22,23,24,25,26,27,28,29,30,31,32,33,34,35,36,37,38,40]. CRT, chemoradiotherapy; ICI, immune-checkpoint inhibitor; SBRT, stereotactic body radiotherapy.

Figure 3 separates stage-based management from possible biomarker-informed choices and research-only strategies. Genomic subtype does not directly route a patient to a required chemotherapy regimen. Unclassified tumors remain eligible for stage-appropriate care. Trial eligibility should use the actual assay and target criteria rather than presumed equivalence between genomic and transcriptional categories. This framework is a decision aid for multidisciplinary discussion, not a validated predictive algorithm.

## 9. Limitations and Evidence Gaps

Diagnostic variability is a major limitation: registry coding, scant biopsies, combined histology, and inconsistent central review can produce different study populations. Retrospective treatment comparisons also differ in stage, performance status, treatment line, and access to molecular testing. Small prospective studies improve ascertainment but remain vulnerable to imprecision and, when nonrandomized, confounding. Pooled observational estimates do not resolve these problems. The narrative selection process introduces additional potential bias and cannot guarantee exhaustive coverage.

Randomized evidence from mixed high-grade neuroendocrine populations, such as JCOG1205/1206, must be distinguished from LCNEC-specific randomized predictive validation [21,22]. In particular, prospective data do not yet establish molecular subtype as a chemotherapy-selection biomarker, routine consolidation immunotherapy after LCNEC chemoradiotherapy, a standard salvage regimen, or PCI. Generalizability to patients with poor functional status, severe comorbidities, or inadequate tissue is especially uncertain.

Biomarker analyses introduce additional challenges. Definitions of “RB1 wild type” may reflect only the absence of a reportable sequence variant, while protein expression and pathway function are not identical measurements. Tumor purity, platform coverage, archival tissue quality, and whether RNA profiling was available can alter subgroup assignment. Studies that exclude inadequate samples may preferentially represent patients with resected or readily accessible tumors. These sources of selection and misclassification should be considered before applying a published subtype association to a patient with a small metastatic biopsy. Negative or inconclusive profiling should not be interpreted as evidence that the tumor lacks relevant biology.

## 10. Conclusions

Pulmonary LCNEC requires integrated morphological and clinical assessment. Surgery and platinum-based chemotherapy remain central, while prospective chemo-immunotherapy observations expand an evidence base still dominated by nonrandomized comparisons. Molecular profiling can inform selected actionable decisions and trial access, but genomic resemblance to SCLC or NSCLC does not establish a preferred regimen. Treatment recommendations should retain their diagnostic, design-related, and patient-selection qualifications.

## 11. Future Directions

International trials should incorporate central pathology review, explicit recording of combined histology, and separate genomic and transcriptional measurements. Predictive validation requires a treatment-by-biomarker interaction, not merely survival differences between molecular groups. Adaptive or collaborative rare-tumor designs could evaluate chemotherapy backbones and ICIs while preserving interpretable comparisons. Serial tissue and circulating DNA may inform evolution, although circulating DNA alone cannot define morphology or transcriptional state. DLL3 and other targets require assay-defined eligibility and clinical response validation. Dedicated relapse studies should include symptom burden, toxicity, quality of life, and CNS outcomes alongside survival.

Trial design should also separate pure LCNEC from combined tumors and distinguish treatment-naïve disease from progression after chemo-immunotherapy. Standardized brain imaging and predefined management of CNS disease would make intracranial outcomes more interpretable. For targeted approaches, biomarker expression should be measured with reproducible assays and linked prospectively to response duration, rather than used only for retrospective subgroup descriptions. Collaborative registries can support feasibility and identify uncommon actionable cases, but should complement, rather than substitute for, prospective comparative evaluation. These priorities would help turn an increasingly detailed molecular taxonomy into decisions that improve outcomes and remain feasible when tissue is limited.

A practical research endpoint is whether obtaining a molecular result changes a decision and improves its outcome. Future studies should record the intended treatment before profiling, the recommendation after profiling, access to the proposed therapy, and reasons for nonimplementation. This would distinguish analytical information from actual clinical utility. Tissue sufficiency, turnaround time, cost, and access to specialist pathology review should also be measured, because a strategy that works only in highly selected research specimens may be difficult to implement in routine advanced disease. Patient-reported outcomes and treatment discontinuation should be captured to assess whether longer disease control is achieved at an acceptable burden. A prospective framework with these features could test the clinical value of precision oncology without assuming that every molecular distinction requires a different drug.

## Figures and Tables

**Table 1:** Epidemiologic and clinical profile of large-cell neuroendocrine carcinoma (LCNEC).

Domain	Current Evidence	Key References
Frequency	Approximately 0.5–1% of lung cancers in contemporary datasets; SEER age-adjusted incidence ~0.3/100,000	[7,8,9,10]
Typical patient	Older adult; strong smoking association; historical male predominance	[7,8,9,11]
Presentation	Advanced disease is common; brain, liver, bone and adrenal metastases are clinically important	[7,8,9,10,11,12,13]
Prognosis	Population-based 5-year OS approximately 16–17%, strongly stage dependent	[7,8,9,10]
Core challenge	Early systemic relapse plus molecular heterogeneity	[3,4,5,14]

Abbreviations and notes: LCNEC, large-cell neuroendocrine carcinoma; OS, overall survival; SEER, Surveillance, Epidemiology, and End Results. Original evidence synthesis for this manuscript; data sources are cited in the table.

**Table 2:** Clinical evidence by study design.

Setting and Design	Findings	Clinical Interpretation
Surgery and adjuvant therapy NCDB/small prospective studies	Surgical outcomes and stage IB chemotherapy association [18,19,20]	Selection bias; routine chemotherapy benefit in stage IA unresolved
Adjuvant JCOG1205/1206 Randomized mixed-histology trial	Cisplatin-irinotecan did not outperform cisplatin-etoposide [21,22]	Includes LCNEC and SCLC; no observation-only arm
First-line chemotherapy Single-arm phase II studies	Cisplatin-etoposide and cisplatin-irinotecan show activity [15,27]	No randomized advanced-LCNEC backbone comparison
Molecular selection Retrospective cohorts	RB1-wild-type association with platinum-gemcitabine/taxanes [30,31]	Not a validated predictive rule; do not generalize to pemetrexed
LANCE Prospective nonrandomized pilot	17 patients; atezolizumab + chemotherapy n = 10, chemotherapy n = 7 [33]	Small groups and nonrandom allocation limit causal inference
Durvalumab + platinum-etoposide Single-arm phase II	12 treated of planned 22; ORR 50%; median PFS 4.4 months; OS 12.8 months [34]	Early closure; descriptive and underpowered
NEJ044 Prospective observational study	77 enrolled/76 treated; approximately 70% 1-year OS [35]	ASCO 2026 abstract; heterogeneous atezolizumab-containing regimens
ICI meta-analysis Predominantly observational evidence	22 studies/3537 patients; pooled ORR 49%; PFS HR 0.51; first-line OS HR 0.72 [36]	Pooled association does not remove confounding
LOGIK2401/NECTAR Phase II protocol and registry	Target n = 30; registry listed Recruiting on 23 October 2026 [37,38]	Planned size is not confirmed enrollment; no efficacy result inferred
Unresectable stage III NCDB/retrospective series	CRT associated with longer OS than chemotherapy alone [24,25,26]	Does not establish optimal concurrent/sequential schedule
Second-line chemotherapy Retrospective LCNEC/possible LCNEC	n = 39; ORR 7.7%; median PFS 3.3 months; OS 8.3 months [40]	Amrubicin predominated; no established standard sequence
CNS treatment Retrospective cohorts	Brain relapse risk and SRS feasibility [10,12,13,25,26,41,42,43]	Individualize local therapy; routine PCI lacks randomized LCNEC evidence

Abbreviations and notes: CRT, chemoradiotherapy; HR, hazard ratio; ICI, immune-checkpoint inhibitor; NCDB, National Cancer Database; ORR, objective response rate; OS, overall survival; PFS, progression-free survival; PCI, prophylactic cranial irradiation; SRS, stereotactic radiosurgery. HRs compare chemo-immunotherapy with chemotherapy. Results are not intended for direct cross-trial comparison. Original evidence synthesis for this manuscript; data sources are cited in the table.

**Table 3:** Biomarkers and therapeutic opportunities.

Biomarker	Possible Clinical Relevance	Evidence Limitation
TP53/RB1 co-inactivation	SCLC-like genomic context [3,30]	Neither diagnostic of SCLC nor a validated chemotherapy selector
STK11/KEAP1/KRAS	NSCLC-like genomic context [3,4,5]	Does not imply sensitivity to every NSCLC regimen
DLL3 expression	Eligibility for target-directed trials [14,39]	Expression is not proof of LCNEC clinical efficacy
FGL1/LAG-3 axis	Biological hypothesis for immune targeting [14]	No validated LCNEC efficacy or selection threshold
SPINK1 expression	Potential investigational vulnerability [14]	Expression evidence; therapeutic benefit unproven
PD-L1/immune phenotype	Additional biological context [32,34,36]	No validated standalone LCNEC selection threshold
Validated actionable driver	Matched therapy or basket-trial discussion [3,5,14]	Variant-specific actionability; often extrapolation from NSCLC

Abbreviations and notes: LCNEC, large-cell neuroendocrine carcinoma; NSCLC, non-small-cell lung cancer; SCLC, small-cell lung cancer. Expression, targetability, and clinical efficacy are distinct evidence levels. Original evidence synthesis for this manuscript; data sources are cited in the table.

## Data Availability

No new patient-level data were generated or analyzed. The evidence discussed is available in the cited publications and trial registries.

## References

[1] Travis W.D., Linnoila R.I., Tsokos M.G., Hitchcock C.L., Cutler G.B., Nieman L., Chrousos G., Pass H., Doppman J. (1991). Neuroendocrine tumors of the lung with proposed criteria for large-cell neuroendocrine carcinoma: An ultrastructural, immunohistochemical, and flow cytometric study of 35 cases. Am. J. Surg. Pathol..

[2] Nicholson A.G., Tsao M.S., Beasley M.B., Borczuk A.C., Brambilla E., Cooper W.A., Dacic S., Jain D., Kerr K.M., Lantuejoul S. (2022). The 2021 WHO Classification of Lung Tumors: Impact of Advances Since 2015. J. Thorac. Oncol..

[3] Rekhtman N., Pietanza M.C., Hellmann M.D., Naidoo J., Arora A., Won H., Halpenny D.F., Wang H., Tian S.K., Litvak A.M. (2016). Next-Generation Sequencing of Pulmonary Large Cell Neuroendocrine Carcinoma Reveals Small Cell Carcinoma–like and Non–Small Cell Carcinoma–like Subsets. Clin. Cancer Res..

[4] George J., Walter V., Peifer M., Alexandrov L.B., Seidel D., Leenders F., Maas L., Müller C., Dahmen I., Delhomme T.M. (2018). Integrative genomic profiling of large-cell neuroendocrine carcinomas reveals distinct subtypes of high-grade neuroendocrine lung tumors. Nat. Commun..

[5] Miyoshi T., Umemura S., Matsumura Y., Mimaki S., Tada S., Makinoshima H., Ishii G., Udagawa H., Matsumoto S., Yoh K. (2017). Genomic Profiling of Large-Cell Neuroendocrine Carcinoma of the Lung. Clin. Cancer Res..

[6] Merola E., Dore M.P., Fanciulli G. (2026). Interventional Clinical Trials in Metastatic Pulmonary Large-Cell Neuroendocrine Carcinoma: A Systematic Review of Prospective, Interventional Trials. Cancers.

[7] Cao L., Li Z.-W., Wang M., Zhang T.-T., Bao B., Liu Y.-P. (2019). Clinicopathological characteristics, treatment and survival of pulmonary large cell neuroendocrine carcinoma: A SEER population-based study. PeerJ.

[8] Deng C., Wu S.-G., Tian Y. (2019). Lung Large Cell Neuroendocrine Carcinoma: An Analysis of Patients from the Surveillance, Epidemiology, and End-Results (SEER) Database. Med. Sci. Monit..

[9] Derks J.L., Hendriks L.E., Buikhuisen W.A., Groen H.J.M., Thunnissen E., van Suylen R.-J., Houben R., Damhuis R.A., Speel E.J.M., Dingemans A.-M.C. (2016). Clinical features of large cell neuroendocrine carcinoma: A population-based overview. Eur. Respir. J..

[10] Kinslow C.J., May M.S., Saqi A., Shu C.A., Chaudhary K.R., Wang T.J.C., Cheng S.K. (2020). Large-Cell Neuroendocrine Carcinoma of the Lung: A Population-Based Study. Clin. Lung Cancer.

[11] Fasano M., Della Corte C.M., Papaccio F., Ciardiello F., Morgillo F. (2015). Pulmonary Large-Cell Neuroendocrine Carcinoma: From Epidemiology to Therapy. J. Thorac. Oncol..

[12] Naidoo J., Santos-Zabala M.L., Iyriboz T., Woo K.M., Sima C.S., Fiore J.J., Kris M.G., Riely G.J., Lito P., Iqbal A. (2016). Large Cell Neuroendocrine Carcinoma of the Lung: Clinico-Pathologic Features, Treatment, and Outcomes. Clin. Lung Cancer.

[13] Zhao Y., Castonguay M., Wilke D., Xu Z., Plourde M., Mulroy L., MacNeil M., Bowes D. (2019). Treatment outcomes and incidence of brain metastases in pulmonary large cell neuroendocrine carcinoma. Curr. Probl. Cancer.

[14] Nassar A.H., Kim C., Adeyelu T., Bou Farhat E., Abushukair H., Rakaee M., Matteson K., Lau S.-F., Takabe Y., Ocejo A. (2025). Integrated molecular and clinical characterization of pulmonary large cell neuroendocrine carcinoma. Nat. Commun..

[15] Le Treut J., Sault M.C., Lena H., Souquet P.J., Vergnenegre A., Le Caer H., Berard H., Boffa S., Monnet I., Damotte D. (2013). Multicentre phase II study of cisplatin–etoposide chemotherapy for advanced large-cell neuroendocrine lung carcinoma: The GFPC 0302 study. Ann. Oncol..

[16] Derks J.L., Dingemans A.C., van Suylen R., den Bakker M.A., Damhuis R.A.M., van den Broek E.C., Speel E., Thunnissen E. (2019). Is the sum of positive neuroendocrine immunohistochemical stains useful for diagnosis of large cell neuroendocrine carcinoma (LCNEC) on biopsy specimens?. Histopathology.

[17] Febres-Aldana C.A., Chang J.C., Ptashkin R., Wang Y., Gedvilaite E., Baine M.K., Travis W.D., Ventura K., Bodd F., Yu H.A. (2022). Rb Tumor Suppressor in Small Cell Lung Cancer: Combined Genomic and IHC Analysis with a Description of a Distinct Rb-Proficient Subset. Clin. Cancer Res..

[18] Raman V., Jawitz O.K., Yang C.-F.J., Voigt S.L., Tong B.C., D’Amico T.A., Harpole D.H. (2019). Outcomes for Surgery in Large Cell Lung Neuroendocrine Cancer. J. Thorac. Oncol..

[19] Iyoda A., Hiroshima K., Moriya Y., Takiguchi Y., Sekine Y., Shibuya K., Iizasa T., Kimura H., Nakatani Y., Fujisawa T. (2006). Prospective Study of Adjuvant Chemotherapy for Pulmonary Large Cell Neuroendocrine Carcinoma. Ann. Thorac. Surg..

[20] Raman V., Jawitz O.K., Yang C.-F.J., Tong B.C., D’Amico T.A., Berry M.F., Harpole D.H. (2019). Adjuvant Therapy for Patients With Early Large Cell Lung Neuroendocrine Cancer: A National Analysis. Ann. Thorac. Surg..

[21] Kenmotsu H., Niho S., Tsuboi M., Wakabayashi M., Ishii G., Nakagawa K., Daga H., Tanaka H., Saito H., Aokage K. (2020). Randomized Phase III Study of Irinotecan Plus Cisplatin Versus Etoposide Plus Cisplatin for Completely Resected High-Grade Neuroendocrine Carcinoma of the Lung: JCOG1205/1206. J. Clin. Oncol..

[22] Kenmotsu H., Niho S., Tsuboi M., Wakabayashi M., Ishii G., Asahina H., Watanabe K., Kiyoshima M., Iwata T., Nakamura Y. (2026). Five-year overall survival in JCOG1205/1206: Irinotecan or etoposide plus cisplatin for resected high-grade neuroendocrine carcinoma of the lung. Lung Cancer.

[23] Lo H., Abel S., Finley G., Weksler B., Colonias A., Wegner R.E. (2020). Surgical resection versus stereotactic body radiation therapy in early stage bronchopulmonary large cell neuroendocrine carcinoma. Thorac. Cancer.

[24] Limonnik V., Abel S., Finley G.G., Long G.S., Wegner R.E. (2020). Factors associated with treatment receipt and overall survival for patients with locally advanced large cell neuroendocrine carcinoma of the lung: A National Cancer Database analysis. Lung Cancer.

[25] Metro G., Ricciuti B., Chiari R., Baretti M., Falcinelli L., Giannarelli D., Sidoni A., Mountzios G., Crinò L., Bellezza G. (2016). Survival outcomes and incidence of brain recurrence in high-grade neuroendocrine carcinomas of the lung: Implications for clinical practice. Lung Cancer.

[26] Prelaj A., Rebuzzi S.E., Del Bene G., Giròn Berrìos J.R., Emiliani A., De Filippis L., Prete A.A., Pecorari S., Manna G., Ferrara C. (2017). Evaluation of the efficacy of cisplatin–etoposide and the role of thoracic radiotherapy and prophylactic cranial irradiation in LCNEC. ERJ Open Res..

[27] Niho S., Kenmotsu H., Sekine I., Ishii G., Ishikawa Y., Noguchi M., Oshita F., Watanabe S.-I., Nakajima R., Tada H. (2013). Combination Chemotherapy with Irinotecan and Cisplatin for Large-Cell Neuroendocrine Carcinoma of the Lung: A Multicenter Phase II Study. J. Thorac. Oncol..

[28] Yamazaki S., Sekine I., Matsuno Y., Takei H., Yamamoto N., Kunitoh H., Ohe Y., Tamura T., Kodama T., Asamura H. (2005). Clinical responses of large cell neuroendocrine carcinoma of the lung to cisplatin-based chemotherapy. Lung Cancer.

[29] Sun J.-M., Ahn M.-J., Ahn J.S., Um S.-W., Kim H., Kim H.K., Choi Y.S., Han J., Kim J., Kwon O.J. (2012). Chemotherapy for pulmonary large cell neuroendocrine carcinoma: Similar to that for small cell lung cancer or non-small cell lung cancer?. Lung Cancer.

[30] Derks J.L., Leblay N., Thunnissen E., van Suylen R.J., den Bakker M., Groen H.J.M., Smit E.F., Damhuis R., van den Broek E.C., Charbrier A. (2018). Molecular Subtypes of Pulmonary Large-cell Neuroendocrine Carcinoma Predict Chemotherapy Treatment Outcome. Clin. Cancer Res..

[31] Derks J.L., van Suylen R.J., Thunnissen E., den Bakker M.A., Groen H.J., Smit E.F., Damhuis R.A., van den Broek E.C., Speel E.-J.M., Dingemans A.-M.C. (2017). Chemotherapy for pulmonary large cell neuroendocrine carcinomas: Does the regimen matter?. Eur. Respir. J..

[32] Eichhorn F., Harms A., Warth A., Muley T., Winter H., Eichhorn M.E. (2018). PD-L1 expression in large cell neuroendocrine carcinoma of the lung. Lung Cancer.

[33] Evangelou G., Trontzas I.P., Gkiozos I., Vamvakaris I., Paraskeva C., Grammoustianou M., Gomatou G., Tsamis I., Vathiotis I., Anagnostakis M. (2024). The Effectiveness of Atezolizumab in Metastatic Large Cell Neuroendocrine Carcinoma of the Lungs: Insights from the LANCE Pilot Study. Biomedicines.

[34] Dudnik E., Nechushtan H., Rotem O., Hanovich E., Wollner M., Boikaner T., Fridman N., Ofer J., Ben-David M.A., Reinhorn D. (2026). Phase II study of 1st-line durvalumab and platinum–etoposide in advanced large-cell neuroendocrine lung carcinoma (aLCNEC). Cancer Immunol. Immunother..

[35] Matsuda S., Asao T., Shukuya T., Nakachi I., Watanabe S., Kishi K., Makiguchi T., Fukuhara T., Harada T., Doushita K. (2026). Chemoimmunotherapy for large cell neuroendocrine carcinoma (LCNEC) of the lung: A multi-institutional prospective study (NEJ044). J. Clin. Oncol..

[36] Tsukada A., Tsurumaki N., Nishimura N., Nokihara H., Izumi S., Hojo M. (2026). Efficacy and safety of immunotherapy in advanced pulmonary large-cell neuroendocrine carcinoma: A systematic review and meta-analysis. Lung Cancer.

[37] Ishii H., Azuma K., Kiyomi F., Yoshimine K., Uchida K., Tobino K., Yamada T., Iwasa T., Kanemura H., Sumi T. (2025). Study protocol of an open-label prospective phase II study of Durvalumab plus Carboplatin and Etoposide in advanced large cell neuroendocrine carcinoma of the lung (LOGIK2401: NECTAR study). Cancer Treat. Res. Commun..

[38] Japan Registry of Clinical Trials LOGIK2401/NECTAR, jRCTs071240054. https://jrct.mhlw.go.jp/en-latest-detail/jRCTs071240054.

[39] Hermans B.C.M., Derks J.L., Thunnissen E., van Suylen R.J., den Bakker M.A., Groen H.J.M., Smit E.F., Damhuis R.A., van den Broek E.C., Ruland A. (2019). DLL3 expression in large cell neuroendocrine carcinoma (LCNEC) and association with molecular subtypes and neuroendocrine profile. Lung Cancer.

[40] Iida Y., Wakuda K., Kenmotsu H., Doshita K., Kodama H., Nishioka N., Miyawaki E., Miyawaki T., Mamesaya N., Kobayashi H. (2024). Efficacy of second-line chemotherapy in patients with pulmonary large cell neuroendocrine carcinoma. Sci. Rep..

[41] Wegner R.E., Hasan S., Williamson R.W., Finley G., Fuhrer R., Colonias A., Karlovits S.M. (2019). Management of brain metastases from large cell neuroendocrine carcinoma of the lung: Improved outcomes with radiosurgery. Acta Oncol..

[42] Kawabe T., Yamamoto M., Sato Y., Yomo S., Kondoh T., Nagano O., Serizawa T., Tsugawa T., Okamoto H., Akabane A. (2016). Gamma Knife radiosurgery for brain metastases from pulmonary large cell neuroendocrine carcinoma: A Japanese multi-institutional cooperative study (JLGK1401). J. Neurosurg..

[43] Popov P., Steindl A., Wolff L., Bergen E.S., Eckert F., Frischer J.M., Widhalm G., Fuereder T., Raderer M., Berghoff A.S. (2024). Clinical characteristics, treatment, and outcome of patients with large cell neuroendocrine carcinoma of the lung and brain metastases—Data from a tertiary care center. Clin. Exp. Metastasis.

